# How Many Is Too Many? On the Relationship between Research Productivity and Impact

**DOI:** 10.1371/journal.pone.0162709

**Published:** 2016-09-28

**Authors:** Vincent Larivière, Rodrigo Costas

**Affiliations:** 1 Université de Montréal, École de bibliothéconomie et des sciences de l'information, C.P. 6128, Succ. Centre-Ville, H3C 3J7 Montréal, Qc., Canada; 2 Université du Québec à Montréal, Centre interuniversitaire de recherche sur la science et la technologie (CIRST), Observatoire des sciences et des technologies (OST), C.P. 8888, Succ. Centre-Ville, H3C 3P8 Montreal, Qc., Canada; 3 Leiden University, Centre for Science and Technology Studies (CWTS), Wassenaarseweg 62A, 2333 AL Leiden, The Netherlands; Universidad de las Palmas de Gran Canaria, SPAIN

## Abstract

Over the last few decades, the institutionalisation of quantitative research evaluations has created incentives for scholars to publish as many papers as possible. This paper assesses the effects of such incentives on individual researchers’ scientific impact, by analysing the relationship between their number of articles and their proportion of highly cited papers. In other words, does the share of an author’s top 1% most cited papers increase, remain stable, or decrease as his/her total number of papers increase? Using a large dataset of disambiguated researchers (N = 28,078,476) over the 1980–2013 period, this paper shows that, on average, the higher the number of papers a researcher publishes, the higher the proportion of these papers are amongst the most cited. This relationship is stronger for older cohorts of researchers, while decreasing returns to scale are observed for recent cohorts. On the whole, these results suggest that for established researchers, the strategy of publishing as many papers as possible did not yield lower shares of highly cited publications, but such a pattern is not always observed for younger scholars.

## Introduction

Over the last few decades, evaluations have become widespread in various spheres of society [1]. Despite being assessed internally through peer review since the second half of the 20^th^ Century, research has, for most of its modern existence, been exempt from external evaluations—thanks to post-WWII economic and scientific growth, as well as the general idea, advocated by Vannevar Bush [2], that science should be free of external interventions. Means of evaluating research and scholars have, however, slowly changed during the 1980s and 1990s, with researchers, administrators, and policy makers gradually incorporating bibliometric indicators in the process. Such quantitative analyses of research activity and impact gained further importance in the 2000s [3], when an increasing set of tools and indicators for assessing individual researcher’s output and impact—such as Google Scholar and the h-index—were developed and made easily available. While in some cases bibliometric assessments were performed to complement peer review in the allocation of research funding—such as the BOF-key in Flanders (Belgium) [4] or the Research Assessment Exercise/Framework in the UK—they have, in other settings, become the only means through which research is assessed and funded [5]. Various publication-based and citation-based funding models can now be found in Australia, Norway, Denmark, Sweden and Finland—and translates as the ‘currency’ through which academic exchanges of tenure, promotion and salary raises are made [6].

The institutionalisation of these evaluations led many researchers to put large emphasis on the number of papers they published. This has led to adverse effects [7–10]. Indeed, like any social group, researchers might be prone to change their behaviour once the rules of the game or what is expected from them become explicit; a phenomenon that could be referred to as the Hawthorne effect [11], and that can be associated with Goodhart’s [12] and Campbell’s [13] laws. As most evaluations and rankings are based on numbers of papers published, this has created incentives for researchers to *publish as many papers as possible*. For instance, in Australia, where publication counts were used without any emphasis on publication venue or citations, researchers have been found to increase the numbers of publications in journals with high acceptance rates and lower impact [14].

In this research evaluation culture of quantity, researchers may have adopted different publication strategies. For example, some researchers might focus on publishing few, high-quality papers—e.g. being more ‘selective’ [15] or ‘perfectionist’ [16], while some others may aim at publishing as many papers as possible, irrespective of their quality—e.g. ‘mass producers’ [16] or ‘big producers’ [17]. The practice of publishing as many papers as possible—often referred to as ‘salami slicing’—has been long discussed in the literature [18–20]. However, only a few authors have analysed the effect of these publication behaviour on citations received, or more generally, the relationship between research output and scientific impact at the individual researchers’ level. For instance, using a sample of 99 male scholars at prestigious US universities, Feist [21] showed that eminence—defined as a mix of peer assessed creativity, visibility and honours received—was most likely associated with scholars who publish a lot of papers, rather than with selective scholars. Similarly, Hanssen and Jørgensen [22] analysed the effect of ‘experience’ on papers’ citations; experience being defined as the author’s previous number of publications. Drawing a sample of papers in transportation research (N = 779) they showed that experience is a statistically significant determinant of individual papers’ citations, although this increase becomes marginal once a certain threshold is met in terms of papers previously published. Supporting this, Bornmann and Daniel [23] have shown, for a small sample of PhD research projects in biomedicine (N = 96), that an increase in the number of papers associated with a project leads to an increase in the total citation counts of the set of papers. Finally, using 74,000 Swedish publications and 48,000 authors for the period 2008–2011, a strong relationship between scholars’ research output and their probability of producing highly cited publications was found, even when fractional counting of papers is used [24].

This paper expands on such previous work, with a larger dataset and over a longer time period. Indeed, using a large dataset of distinct disambiguated researchers (N = 28,078,476) who published at least one paper during the 1980–2013 period, this paper aims to better understand the relationship between publication activity and scholarly impact. More specifically, it aims to answer the following research question: what is the relationship between research productivity and scholarly impact? Is the share of scholars’ top papers increasing, stable, or declining, as their research productivity increases? In other words, can scholars be too productive? A good analogy for this is archery: if an archer throws one arrow, what is the probability that it hits the center of the target? Does an increase in the number of arrows thrown leads to an increase in the proportion arrows hitting the center of the target? Our working hypothesis is that authors with higher number of papers would also publish a higher proportion of top cited papers. Such hypothesis would be in agreement with Merton’s theory of cumulative advantage [25], and supported by the empirical work in the sociology of science [16]. Similarly, in a Bourdieusian framework, the main goal of a researcher is to increase its rank in the scientific hierarchy and gain more scientific capital [26]. If publishing a high number of scientific papers and being abundantly cited are the ways through which researchers can reach this goal, then they will adapt their behaviour to reach these evaluation criteria.

## Methods

This paper uses Thomson Reuters’ Web of Science (WoS) database for the period 1980–2013. Only journal articles are included. Given that the unit analysed in this paper are individual researchers, we used the author disambiguation algorithm developed by Caron & van Eck [27] to identify the papers authored by individual researchers. On the whole, the algorithm managed to attribute papers to 28,078,476 individuals who have published their first paper between 1980 and 2013. In order to assess differences across disciplines, and to take into account scholars’ publication and citation patterns [28]—we categorized researchers into four disciplinary domains based on their publication venues: 1) law, arts, and humanities, 2) medical and life sciences, 3) natural sciences, and 4) social and behavioral sciences. Such categorization was based on a reclassification of WoS Subject Categories into four main disciplinary categories.

Three methods were tested to take into account the fact that researchers might publish in more than one of the four broad disciplinary domains. A first method was to consider author-domain combinations [29] as distinct entities, and to divide a researcher in as many entities (MAX = 4) as there are domains in which his or her papers were published. The disadvantage of such method for this paper is that it splits those who have published in more than one broad field into different “researchers,” which, in turn, reduces their total research output. It also increases the number of scholars analysed by 12.2% to 31,490,527. A second method was to use the broad discipline in which a scholar was the most active in terms of number of papers as his/her main discipline, and assign all of his/her papers into that discipline. In the case of a tie—an author who has published the same maximum number of papers in two fields—the field of the researcher was chosen randomly among the fields with the highest number of papers. Finally, a third method was only to count the researcher in the field in which he/she has the highest number of papers, and to restrict the papers analysed to those in that discipline. Although all three methods yielded similar results because of the large numbers involved, we chose the second method, as it includes all the research output of a scholar into the field that is most likely to be her/his main one.

As our aim is to assess researchers’ contribution to papers that have the highest impact, we isolated the top 1% most cited papers published each year for each discipline (normalised by WoS subject categories). Citations were counted until the end of 2013, and any overlap between the groups of citing and of cited authors (self-citations) were excluded. Hence, the paper only focuses on ‘external citations’ as these are the most relevant for evaluative purposes [30].

**Fig 1:**
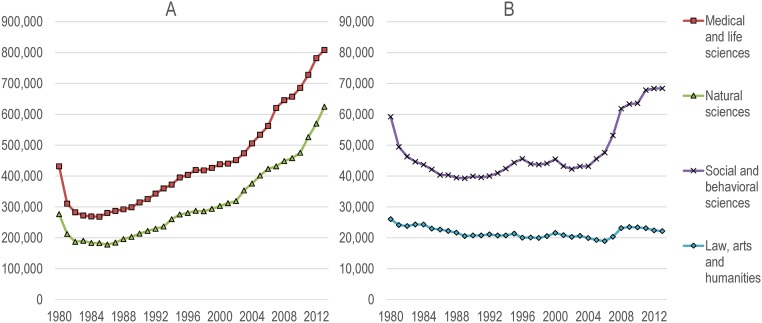
Number of disambiguated researchers, by year of first publication and broad field.

Fig 1 presents the number of scholars analysed in the paper, according to their year of first publication. The fact that the database starts in 1980 explains why we observe a decrease in the number of new scholars for the first few years of the database (as all 1980 scholars are *de facto* “new” scholars). For disciplines of the natural and medical sciences (Panel A), we observe a steep increase of the number of new scholars from the mid-1980s onwards. For the social and behavioral sciences (Panel B), the early 1980s decrease is followed by a relatively stable number of new authors, until the early 2000s when the number of new authors increase from about 40,000 to 70,000 annually. For law, arts, and humanities, the number of new authors is relatively stable, if not slightly decreasing, throughout the period.

S1 Table presents the main descriptive values for the dataset of scholars studied, focusing on the two main variables under study (i.e. the total number of papers and the share of top 1% articles). As one could expect, differences in research productivity are observed across disciplines, a pattern already documented by Ruiz-Castillo & Costas [29]. Similarly, some differences in the proportion of top 1% publications can also be observed across disciplines and cohorts. Interestingly, we observe an increase in the number of active scholars between cohort 1981–1985 and 2009–2013 in all disciplines but Law, arts and humanities, which suggest that the coverage of the WoS has, in those disciplines, actually decreased over the last 30 years.

## Results

**Fig 2:**
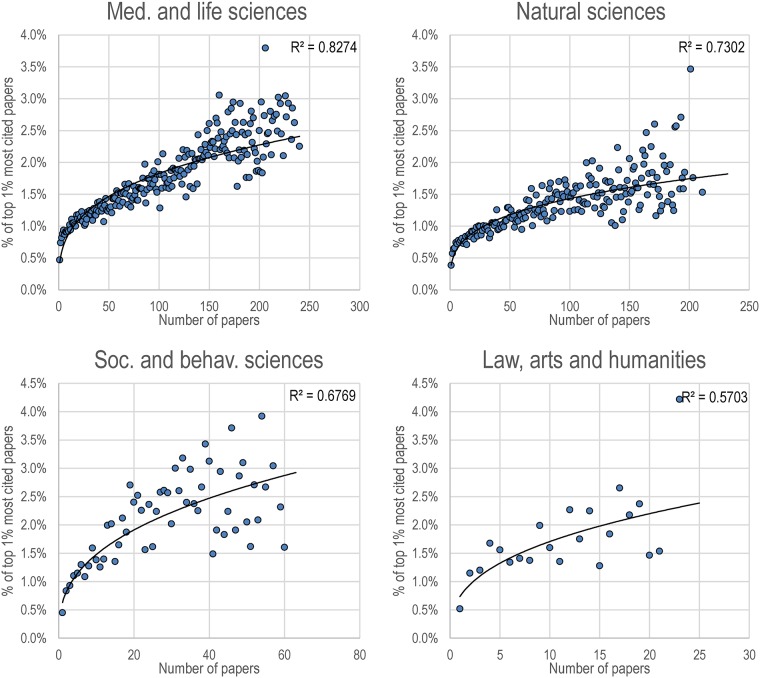
Proportion of top 1% most cited papers (y axis), as a function of the number of papers published (x axis), for the cohort of researchers who have published their career number between 1981 and 1985, by domain. Only classes of numbers of papers with 30 researchers or more are shown. Power trendlines and R2 are were obtained using the Excel software.

Fig 2 presents, for the oldest cohort studied—researchers who have published their first paper between 1981 and 1985—the relationship between the number of papers throughout their career and the proportion of those papers that made it to the top 1% most cited. For any specific number of papers, the expected value of top 1% papers is, as one might expect, 1%. For each of the four broad domains, authors with very few papers are, on average, much less likely to publish high shares of top 1% most cited papers. But, even more importantly, we also see that for each domain, there is an increase in the proportion of top 1% most cited papers as researchers’ output increase. The strength of the relationship, however, varies by domain, with a stronger relationship in medical and life sciences (R^2^ = 0.83) followed by natural sciences (R^2^ = 0.73), social and behavioral sciences (R^2^ = 0.68) and then by the social sciences and law, arts and humanities (R^2^ = 0.57).

More specifically, medical and life sciences scholars with less than 10 papers generally publish less than 1% of their papers in the top 1% most cited groups of papers, the share of top papers increases with productivity, and reaches values between 2% and 3% for scholars who published more than 200 papers. In natural sciences, the increase is not as fast, with scholars who, roughly, publish less than 45 papers still being less likely to publish a high share of top papers. Along these lines, the results are also more scattered, with the group of scholars publishing 150 papers or more obtaining shares of top 1% most cited papers between 1% and 2.5%. In the social and behavioral sciences, very fast increase is seen in terms of shares of top cited papers: researchers with 4 papers or more already *punch above their weight*, and between 1.5% and 4% papers from researchers who published between 40 and 60 papers were in the top 1% most cited papers. Similar trends can be found in law, arts and humanities, with scholars who have published more than one paper having a share of top papers above average, and scholars with 10–20 papers obtaining a share of top papers between 1.3% and 2.6%.

**Fig 3:**
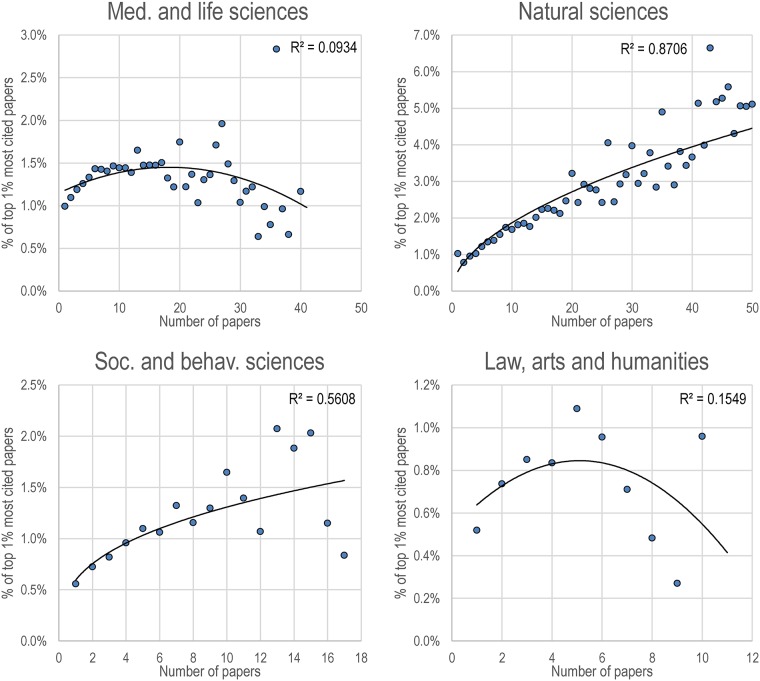
Proportion of top 1% most cited papers, as a function of their number of papers published, for the cohort of researchers who have published their first paper between 2009 and 2013, by domain. Only classes of numbers of papers with 30 researchers or more are shown. Power trendlines and R2 are used for natural sciences and social and behavioral sciences, while 2nd order polynomials are used for medical and life sciences, and law, arts and humanities.

When the subset of younger researchers who have published their first paper between 2009 and 2013 is considered, different patterns are observed (Fig 3). For medical and life sciences, there is an increase in the share of highly cited publications—although with percentages that are lower than those observed for the oldest cohort—slightly after 15 publications, when decreasing returns to scale are observed. And contrary to the older cohort of scholars from this domain, it is those with higher numbers of papers (>30) that obtain lower shares of top papers. For natural sciences, the trend is similar to that obtained for the older cohort, with larger shares of top papers associated to higher levels of research production. In this domain, scholars with high levels of production (e.g. >40 papers) reach shares of top papers that are sometimes between 5 and 7 times world average. For both social and behavioral sciences, and law, arts and humanities, we observe increases in the proportion of top papers as output rises but, in a manner similar to medical and life sciences researchers, decreasing returns to scale (i.e. lower shares of top papers for higher levels of production) are observed.

An important characteristic of this cohort is that it got socialized to research recently—when the evaluation culture was more present—which might make them prone to publish as much as possible. However, the drop in the share of top papers observed in each domain except natural sciences suggests that these academically-younger scholars struggle to keep impact high once a certain threshold is met. This might be due to the fact that these scholars have not yet secured permanent or tenure positions and, thus, might feel that they cannot be as selective as older scholars

**Fig 4:**
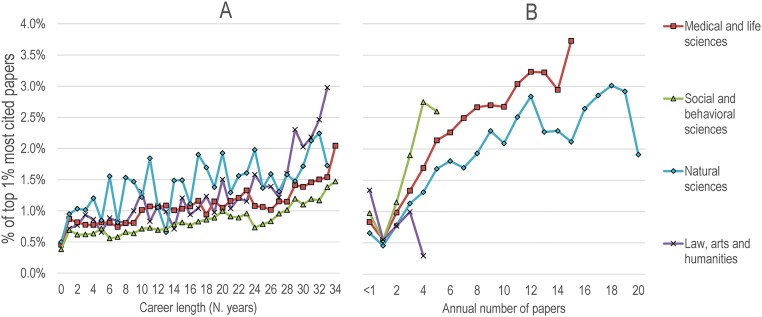
Proportion of top 1% most cited papers for all cohorts of researchers, by domain. A) As a function of career length, B) As a function of their annual number of papers (rounded). Only groups with 30 researchers or more are shown.

In order to take into account scholars’ various career paths, we compiled the proportion of top 1% most cited papers as a function of career length (Fig 4, panel A) for all cohorts combined (Fig 4, panel B). Career length analysis shows that longer careers are associated with a higher proportion of papers published in the top 1% most cited, irrespective of the domain. More specifically, authors with very short careers—which account for the majority of scholars and are likely to be occasional or transient authors who have performed master or doctorate degrees without remaining in research—have systematically lower shares of top papers. As career length increases, shares of top papers increase slightly for natural sciences and medical and life sciences, and at a faster pace for social and behavioral sciences as well as for law, arts and humanities. For all domains but the latter, the longest career length is associated with the highest proportion of top 1% most cited papers. Along these lines, annual numbers of papers are also associated with higher levels of scientific impact. While the positive relationship between the two variables is quite clear for social and behavioral sciences, we observe a flattening of the curve at about 10 papers for medical and life sciences and natural science followed, for the latter field, by an increase in top papers for annual numbers of papers between 30 and 50. Law, arts and humanities, however, follows a different pattern, with scholars having less than one paper/year obtaining the highest proportion of top papers. Although not shown, similar patterns were obtained when only looking at specific cohorts.

## Discussion and Conclusion

Previous research has shown that, in many contexts, the focus on quantitative indicators for research evaluation has had adverse effects [7]. This paper aimed to provide an original analysis of one of these: *to publish as much as possible*. Our results have shown that, for older researchers, the higher the number of papers published, the more likely those ended up being amongst the most cited papers of their discipline. For younger scholars, however, such relationship could only be observed in the natural sciences and, to a certain extent, in the social and behavioral sciences. For the two other broad domains, higher scientific output was associated with decreasing shares of highly cited publications.

Several factors can contribute to the explanation of this pattern. For instance, authorship criteria [31] and co-authorship patterns [32] are different in disciplines of the natural sciences and of medical and life sciences than in the social and behavioral sciences and law, arts and humanities, which might explain why senior researchers are more likely to contribute to many papers (and many top cited ones). Similarly, while this study takes age of academics into account—which has been shown to be a key driver of scholarly output and impact [33, 34]—there are several other factors that have been shown to affect research output and impact, such as interdisciplinary, gender, funding, country, and database used [28, 30, 35–38]. The thorough exploration of all these factors is obviously beyond the objectives and possibilities of this paper, and further research should help to clarify how these factor interact and play a role in the patterns observed here.

From a theoretical point of view, these results conform to the Mertonian theory of cumulative advantages [25]: the higher the number of papers an author contributes to, the more he/she gets known and, hence, is likely to further attract citations. In Bourdieusian terms [26], the more an author publishes and accumulates citations in a domain, the more this capital will yield additional papers and citations. The relationship could also be in the other direction, as authors with a lot of scientific capital might have more opportunities to contribute to papers (e.g. through collaboration, funding, etc.). Still, the results show that highly productive authors, on average, also contribute to more highly cited papers; the fact that this is not consistently observed for younger researchers might be due to the fact that this age group has not had enough time to *stratify* [16] itself into different categories of scholars. Finally, from a practical point of view, the interdependencies between absolute indicators—such as number of publications—and relative indicators—such as the proportion of top cited papers—reinforce the idea of research performance as a multidimensional concept [39], difficult to measure by a reduced set of indicators. This supports the idea of contextualize research performance as a complex ecosystem of different types of scholars, activities, abilities and relationships [40], as well as the idea raised in the *Leiden Manifesto* [41] that individuals are best assessed on a qualitative judgment of their portfolio, combining several indicators and information about their activities.

## Supporting Information

S1 Table Main descriptive values for scholars covered by the analysis. (DOCX)
