# Supplementary material for: How Many Is Too Many? On the Relationship between Research Productivity and Impact
Source: PLoS One. 2016 Sep 28;11(9):e0162709. doi: 10.1371/journal.pone.0162709 (PMC5040433; doi:10.1371/journal.pone.0162709)
Supplement: S1 Table — (DOCX) [file pone.0162709.s001.docx]

**S1 Table. Main descriptive values for scholars covered by the analysis**

| **Cohort/Dataset** | **Discipline** | **N.**  **scholars** | **Avg.**  **prod.** | **Stdev.**  **prod.** | **Max**  **Prod.** | **Min**  **prod.** | **Mean**  **% top**  **papers** | **Stdev.**  **% top**  **papers** | **Max**  **% top**  **papers** | **Min**  **% top**  **papers** |
| --- | --- | --- | --- | --- | --- | --- | --- | --- | --- | --- |
| Total dataset | LAW, ARTS AND HUMANITIES | 738474 | 1.43 | 2.49 | 330 | 1 | 0.58% | 6.51% | 1 | 0 |
|  | MEDICAL AND LIFE SCIENCES | 15108319 | 3.70 | 13.56 | 1769 | 1 | 0.93% | 7.97% | 1 | 0 |
|  | NATURAL SCIENCES | 10605252 | 3.90 | 17.34 | 2230 | 1 | 0.76% | 7.11% | 1 | 0 |
|  | SOCIAL AND BEHAVIORAL SCIENCES | 1626431 | 2.17 | 5.70 | 725 | 1 | 0.60% | 6.07% | 1 | 0 |
| Cohort 1981-1985 | LAW, ARTS AND HUMANITIES | 119566 | 1.58 | 3.39 | 330 | 1 | 0.64% | 6.79% | 1 | 0 |
|  | MEDICAL AND LIFE SCIENCES | 1403170 | 5.93 | 21.92 | 1462 | 1 | 0.62% | 6.11% | 1 | 0 |
|  | NATURAL SCIENCES | 956486 | 6.12 | 25.96 | 2065 | 1 | 0.51% | 5.44% | 1 | 0 |
|  | SOCIAL AND BEHAVIORAL SCIENCES | 226244 | 2.51 | 7.73 | 515 | 1 | 0.61% | 6.08% | 1 | 0 |
| Cohort 2009-2013 | LAW, ARTS AND HUMANITIES | 114567 | 1.22 | 0.78 | 30 | 1 | 0.55% | 6.35% | 1 | 0 |
|  | MEDICAL AND LIFE SCIENCES | 3662268 | 1.57 | 1.74 | 185 | 1 | 1.04% | 8.86% | 1 | 0 |
|  | NATURAL SCIENCES | 2654547 | 1.78 | 5.76 | 656 | 1 | 1.02% | 8.62% | 1 | 0 |
|  | SOCIAL AND BEHAVIORAL SCIENCES | 331538 | 1.42 | 1.24 | 59 | 1 | 0.61% | 6.46% | 1 | 0 |
